# The pleasantness of sensory dissonance is mediated by musical style and expertise

**DOI:** 10.1038/s41598-018-35873-8

**Published:** 2019-01-31

**Authors:** Tudor Popescu, Monja P. Neuser, Markus Neuwirth, Fernando Bravo, Wolfgang Mende, Oren Boneh, Fabian C. Moss, Martin Rohrmeier

**Affiliations:** 1 Institute for Art History and Musicology, TU Dresden, Dresden Germany; 2 Department of Psychiatry and Psychotherapy, Eberhard Karls University Tübingen, Tübingen, Germany; 3 Digital Humanities Institute, École Polytechnique Fédérale de Lausanne, Lausanne, Switzerland; 4 Department of Music, University of California, Berkeley, USA; 5 Department of Neurology, Medical University of Vienna, Vienna, Austria

**Keywords:** Cortex, Human behaviour

## Abstract

Western musical styles use a large variety of chords and vertical sonorities. Based on objective acoustical properties, chords can be situated on a dissonant-consonant continuum. While this might to some extent converge with the unpleasant-pleasant continuum, subjective liking might diverge for various chord forms from music across different styles. Our study aimed to investigate how well appraisals of the roughness and pleasantness dimensions of isolated chords taken from real-world music are predicted by Parncutt’s established model of sensory dissonance. Furthermore, we related these subjective ratings to style of origin and acoustical features of the chords as well as musical sophistication of the raters. Ratings were obtained for chords deemed representative of the harmonic language of three different musical styles (classical, jazz and avant-garde music), plus randomly generated chords. Results indicate that pleasantness and roughness ratings were, on average, mirror opposites; however, their relative distribution differed greatly across styles, reflecting different underlying aesthetic ideals. Parncutt’s model only weakly predicted ratings for all but Classical chords, suggesting that listeners’ appraisal of the dissonance and pleasantness of chords bears not only on stimulus-side but also on listener-side factors. Indeed, we found that levels of musical sophistication negatively predicted listeners’ tendency to rate the consonance and pleasantness of any one chord as coupled measures, suggesting that musical education and expertise may serve to individuate how these musical dimensions are apprehended.

## Introduction

Western music exhibits a large variety of vertical sonorities, ranging from common chords such as major, minor, diminished and seventh chords, and their respective inversions; to numerous sonorities featuring suspensions, augmentations and added dissonances. There are detailed music-theoretical accounts that derive such complex sonorities from simpler prototypes (e.g. refs^1,2^). However, when considering such phenomena in the stylistic context in which they occur (i.e., in ecological music), distinctions such as “consonant” and “dissonant” no longer appear straightforward. Do we even mean the same thing by “dissonance” when we speak about a minor second, a tritone, a fourth suspension, a dominant seventh chord, a whole-tone sonority, a bitonal chord or a serialist tone cluster? Do these sonorities necessarily have a negative valence? Do our judgements of consonance/dissonance and valence reflect intrinsic features, or does their attribution rather depend on their contextual embedding? Research on the perception of consonance and dissonance has to date mostly focussed on simplified, abstract chord representations, but it has a blind spot about phenomena that occur “in the wild”. The present study addresses this lacuna, considering chords from Classical, Jazz, and Avant-garde music.

Throughout the history of music, theorists have proposed rules determining which tones should be preferred or avoided when building composite sonorities^3^. These rules have been formulated in various ways, but tended to gravitate around the concepts of dissonance and consonance. Among the earliest intuitions on the consonance of musical intervals was the Pythagorean theory that such intervals are produced by vibrating strings divided according to simple numerical ratios – 2:1 (the *octave*), 3:2 (the *perfect fifth*) or 5:4 (the *major third*)^4^. On the same grounds, intervals not meeting this criterion, such as 45:32 (the *tritone*), were for a long time considered “undesirable” in composition. As a note on terminology, by *interval* we understand – in conformance with standard music theory – a combination of two notes, sounded either simultaneously (harmonic interval) or in succession (melodic interval); whereas by *chord* we understand a combination of three or more notes sounded simultaneously, and by *sonority* any sound composed of distinct pitches, whether or not this sound can be derived from a root and stacking of thirds.

Despite the fact that the classification system changed over historical periods, the association between the concept of consonance – as construed in music theory and in composers’ practice – and that of liking (pleasantness) endured throughout music history. Indeed, Grove Music Online^5^ even defines consonance and dissonance *in terms of* pleasantness and unpleasantness, respectively. That the consonance/dissonance status of various intervals has not stayed constant over time, and that the role of consonance/dissonance itself varies between cultures (e.g. ref.^6^), suggests that these qualia cannot simply be explained by mere reference to physical or psychoacoustical properties; they are inextricably linked with subjective and cultural dimensions.

While Pythagorean theory was limited to only pairs of consecutive notes (melodic intervals), chords made up of simultaneous combinations of three or more notes required more complex models, which were only developed centuries later. 19th-century German psychophysicists, most notably von Helmholtz, proposed that the dissonance of chords made of several complex notes (as opposed to pure tones) may be associated with a sensory quale referred to as *roughness*^7^. Roughness is the perceived manifestation of a psychoacoustic phenomenon known as *beating*, which describes the interaction between a sound’s various single-frequency components; as these components go in and out of phase with each other, the combined sound is amplitude-modulated, as its waveform waxes and wanes^7,8^. Sounds are said to manifest *sensory dissonance* when beating occurs, and likewise to be *consonant* in its absence (that is, for low *roughness* or high *smoothness*). Experimental evidence suggests a negative correlation between sensory dissonance and liking (*pleasantness*), at least in Western participants (e.g. refs^9–11^). This consistent association has prompted some authors to prefer the term *sensory irritation* to *sensory dissonance*^12^, which places the negative connotation in the psychoacoustical realm.

Several complex models exist that derive a chord’s roughness value based on the frequencies (amplitude spectra) of its individual notes^13–17^. These models propose a mapping of the subjective concept of dissonance onto the objective dimension of roughness. One prominent model has been proposed by Hutchinson & Knopoff^14^ – itself an extension of Plomp and Levelt’s^8^ beating-based model. This model, as implemented by Parncutt^18^, has received some detailed empirical validation (e.g. ref.^19^) and has also been explored in relation with a simplified local context of musical tension^20^. However, it has so far only been evaluated with a small number of basic chord types, which does not bring out the full spectrum of sonorities that appear “in the wild”.

An alternative theoretical account of dissonance is based on Carl Stumpf’s concept of tonal *fusion*^21^. The degree of fusion present in a chord is determined by the combined partials (harmonics) of its component tones: if these are harmonically arranged relative to each other, the overall frequency spectrum of the chord resembles that of a single complex tone, and consequently the chord is perceived as a unity^22^. Tonal fusion as a concept is thus closely related to that of harmonicity. Stumpf insisted that it is fusion that forms the basis of dissonance, rather than beating. In fact, the phenomena of harmonicity and beating are not independent; rather, in a given sonority, one occurs at the expense of the other: *beating* partials reduce the degree of *fusion*. Therefore, the two accounts are not mutually exclusive in explaining dissonance; nevertheless, only the preference for harmonicity, and not also that for beating, predicted listeners’ preference for consonant over dissonant chords, as was shown in studies by McDermott *et al*.^10,23^. In addition, in these studies preference for harmonicity itself correlated positively with musical expertise. Finally, it is noteworthy that attempts linking the pleasantness of the various intervals to an acoustic basis, whether beating- (smoothness-) or fusion-based, date to psychological studies from the 1920s. Although based on data from very few participants, Brues’^24^ pleasantness ratings were not well predicted from fusion ratings, while Guernsey’s^25^ pleasantness ratings did closely follow those for smoothness (i.e., same interval ranking for both measures). That this held only for the musically untrained listeners suggests that dissonance perception is mediated by a listener’s musical background, in concordance with the McDermott *et al*. studies.

So far, we have focused on the psychoacoustical bases of dissonance. However, the perception of complex sonorities also depends upon how psychoacoustics interacts – on the listener-side – with acquired implicit knowledge, enculturation and musical experience (e.g. refs ^26,27^). Considering these factors in their totality raises the broader topic of the objective foundation of consonance preference, and its innate vs acquired nature – the debate over which is far from settled. An important question in this debate is when or to what degree consonance preference emerges naturally. While a bias for consonance is observed in some avian species and non-human primates^28–30^, studies on infants still struggle to converge in proving either the presence or the absence of an intrinsic preference for consonance in humans^31–36^. Some recent studies have adopted a computational approach to consonance, whose defining features – in their Western conceptualisation – have been replicated as emerging non-linear properties of dynamical complex systems^37^ and of Hebbian-learning neural-network simulations^38^, thus supporting the idea of consonance preference as a “natural” property. Finally, studying the neural correlates of auditory perception in humans reveals evidence of a distinct brain basis for consonance and dissonance. For instance, listening to consonant vs dissonant chords is associated with distinct electroencephalographic (EEG) signatures^39^, spatially organised along the auditory cortex^40^, and which are modulated by musical expertise^41^. Furthermore, correlations have been found between measures of consonance preference and the structure^42^ and function^43^ of brainstem nuclei, in particular of the inferior colliculus. A study using functional magnetic resonance imaging (fMRI) has found deactivation of inferior colliculus due to dissonance, in proportion to individual consonance preference^44^; this suggested that subjective consonance preference is underlain by the physiological sensitivity to dissonance of the subcortical auditory system, a sensitivity that has been shown to already be present at birth^33^. To summarise, there is a clear neurophysiological basis to dissonance perception, which serves to counterbalance claims about the role of culture that will be discussed next.

If consonance perception, rather than being innate in humans, were to be largely governed by Western musical enculturation, then non-enculturated listeners would be expected to lack a preference for it. This hypothesis is partially supported by the quoted study of McDermott *et al*.^6^, which found that members of a remote Amazonian tribe (Tsimané) rated consonant and dissonant chords as equally pleasant. Despite certain methodological caveats^45^, this study raised the intriguing hypothesis that “consonance preferences can be absent in cultures sufficiently isolated from Western music, and are thus unlikely to reflect innate biases or exposure to harmonic natural sounds.” That preference for consonance varies with exposure to Western culture, and the existence itself of different musical cultures are not, however, sufficient to conclude that consonance/dissonance perception does not also have a shared – and non-arbitrary – biological basis. Such a basis also likely exists for other features of music perception and production, some of which have been proposed as candidates for cross-cultural musical universals^46–48^, with some authors even arguing that musical cultures are but elaborations of only a handful of such universal traits^49^. Coming back to McDermott *et al*.^6^ however, the generality of its conclusion has been challenged^45,50^, and the debate surrounding the relative roles played by biology and culture – and by their interaction – in dissonance perception has by no means been put to rest. To summarise, evidence as divergent as that reviewed above cumulatively advocates an understanding of music as a “biocultural phenomenon”^51^ and suggests that the perception of consonance and dissonance is grounded in psychoacoustics but mediated (and possibly overridden) by culturally acquired preferences.

As for the interaction between consonance and liking, several studies have examined how this too is affected by the interplay of nature and nurture. As noted by Plomp and Levelt^8^, while musicians typically make a clear distinction between pleasantness and consonance, for less musical individuals the two concepts are barely distinguishable. A similar conclusion was drawn by Roberts^52^, who asked musicians and non-musicians to rate isolated chords in terms of consonance and pleasantness. Previously described effects of chordal properties on ratings were confirmed: increased consonance and pleasantness ratings were obtained for major chords, followed by minor, diminished and augmented; for chords in root position versus inversions; and for chords in equal temperament versus other types of tuning. However, these effects interacted with musical training, in that this hierarchy was only evident for musicians. By contrast, in a more recent study by Lahdelma & Eerola^53^, the top preference ratings obtained for mildly dissonant chords (minor ninth, major ninth and minor seventh) and held across the range of participants’ musical sophistication. To summarise, the exact way in which musical expertise mediates between consonance perception and liking (pleasantness) appears to be inconsistent in studies to date.

As a final component affecting the relationship between consonance and pleasantness, we consider the issue of musical style. Despite the absence of empirical evidence on this issue, we hypothesise that the interaction between consonance and pleasantness ratings of a chord will systematically also depend on the musical “style” of origin and the aesthetic ideals associated with it, even if the latter information is not disclosed to the participants. For instance in jazz, chords that might be described as “pleasant” are likely to be dissonant in any of the senses described above; indeed, the inclusion of dissonances is an intrinsic part of that style’s vernacular^54^. Thus, we share Zatorre’s view that, “rather than mapping onto a simple pleasantness dimension, dissonance and consonance may be better thought of as ways to manipulate sound expressively, and thereby engender emotions or moods”^50^.

With this study, we aimed to investigate the relations between roughness (as a measure of sensory dissonance) and pleasantness across isolated chords of different musical styles (Classical, Jazz, Avant-garde and Random/Control). In particular, we examined the extent to which Parncutt’s model of dissonance predicts the participants’ ratings of “real-world” chords of these styles.

## Methods

### Participants

Participants consisted of 30 healthy volunteers (16 females, 14 males; mean age 24.1 years, range 18–48 years). The only selection criterion was normal functioning of the sense of hearing (by self-report); musical ability in the sample was heterogeneous. All participants gave their written, informed consent, in accordance with the Declaration of Helsinki. The experimental protocol was in concordance with the ethics protocols of the University of Tübingen, and was approved by this institution’s Ethics Committee.

### Stimuli

Twenty different chords (henceforth referred to as “exemplars”) were selected by five experts in music theory in each of four categories (henceforth referred to as “styles”) defined by the Classical, Jazz and Avant-garde (“New Music”) styles; plus a random control category of computationally generated chords. For each of the first three styles, chords originated from ecological contexts and were deemed to be characteristic of the tonal language of their respective style. Classical chords were taken from works by composers of the common-practice period such as Mozart, Haydn, and Mendelssohn. Avant-garde composers included Boulez, Feldman and Scelsi. Jazz chords were selected from a book with transcriptions of classic original Jazz piano recordings (Leonard, 2004). All chords’ musical notation and provenance details can be found in the Supplementary Information.

Random chords were computationally created as controls, and meant to correspond to a “null hypothesis” of no systematic sonic preference towards either consonance or dissonance. Random chords were always made up of five (the median of all other chords’ note-numerosity) notes sampled randomly from a uniform distribution across the chromatic scales in the range D2 to B5.

Each stimulus consisted of a single isolated chord, made up of four to eight notes spanning an overall equal-tempered range from E1 to C7. It was not intended to keep the number of notes per chord constant across or within styles, as this would have greatly constrained the representativeness of the stimulus selection, as described above. All stimuli were constructed using the software packages Sibelius v7.5 and Pro-Tools v12 (both by Avid Technology), and rendered as audio files in the Kontakt player plugin v10, with a 3-second duration per chord, using a grand piano sound with natural fade-out. The sheet music for all chords, as well as the audio files, can be found in the Supplementary Materials.

Stimulus selection for this study aimed for *representative* exemplars from each style. Thus, rather than being a systematic or exhaustive process, the selection was made on the basis of knowledge of the literature of the different styles under investigation. We selected chords based on the relative base rate (frequency of occurrence) of chord types within each style, but also ones that were subjectively “interesting” in their sonic qualities. That is, chords that, in their deviation from what could be construed as the most characteristic chord of a style, provided a “differentia specifica” for each style. Thus, we did not, for instance, include major and minor chords for Classical, despite their frequency in that style. This selection procedure aimed to afford a *representative* and wide range sample of chords in each of the styles.

### Procedure

Participants were tested individually in experimental booths at the Institute of Psychology, University of Tübingen. Participants first filled out a short questionnaire with basic demographic data, before proceeding to the main (rating) task.

The rating task was programmed and administered in PsychoPy v1.83.04^55^. Each of the 80 unique stimuli (4 styles × 20 exemplars per style) was presented twice, for a total of 160 ratings (trials) per participant; breaks occurred between blocks of 40 trials. The overall duration of the task was around 40 minutes. The chords’ presentation order was randomised across the entire pool of exemplars and styles, separately for each of the two repetitions. For each trial, after hearing the chord, participants were asked to rate it for pleasantness and roughness, by positioning a slider along two 7-step Likert scales. The top axis was labelled from “−3: unpleasant” to “+3: pleasant” (German: *unangenehm*, *angenehm*), the bottom axis from “−3: strong roughness” to “+3: weak roughness” (German: *starke Reibung*, *schwache Reibung*). There was no time limit for responding, and there was no default (e.g. zero) rating initially selected at the start of a trial. To avoid any potential carry-over effects between consecutive trials, the presentation of each chord was preceded by a 3-second (in- and out-faded) recording of sea waves, a sound spectrally similar to white noise and thus harmonically neutral (cf. ref.^11^). Stimuli were presented over T.bone HD 800 headphones, at a volume chosen by each participant at the start of the task. The task instructions encouraged use of the entire range of the rating scales; and emphasised that the two ratings, while possibly feeling as if tightly linked, should nonetheless be given independently and without extended contemplation. Participants were not aware of the existence of the four categories to which a stimulus belonged. “Roughness” was explained in terms of the compatibility between the constituent tones of the chord (high compatibility = low roughness). The rating task began with a practice block of 4 trials. In order to have a measure of the participants’ musical background and education, we used the German-language version of the GMSI questionnaire (the Goldsmiths Musical Sophistication Index^56^).

### Analyses

To evaluate the ability of the Parncutt^18^ model to predict roughness ratings, for each style ratings from across all 30 participants and for each of the style’s 20 exemplars, were concatenated into a single vector; and a Pearson correlation was computed *across*-participants against the corresponding model values for each exemplar. An additional set of correlations was computed to check whether GMSI scores can predict *within*-participant *R*s (interpretable as goodness-of-fit for Parncutt’s model).

For each trial, we defined a measure that we term the *sensory decoupling ability* (SDA), computed as the absolute value of the difference between the pleasantness and the smoothness rating of that chord; *smoothness* being simply the *roughness* rating “flipped” with respect to the 7-point maximum of the Likert scale. Based on the hypothesis that, for most people, consonance and pleasantness will be tightly linked, a small SDA would result from the (expected) association between a high pleasantness rating and a high consonance (i.e. weak roughness) rating, while conversely, a high pleasantness rating coupled with a low consonance (high roughness) rating would lead to a high SDA for the current trial. Values for this measure were then averaged in the same way as for the participant-given ratings, i.e. across both repetitions of a given exemplar, and across all exemplars of a given style. Pearson correlations were then computed across-participants between GMSI and SDA, both within and across styles. Using Spearman’s (non-parametric) rank-based correlation coefficient instead of Pearson’s did not change the reported results qualitatively. Wherever required by the statistical procedures employed, normality assumptions were checked using the Kolmogorov-Smirnov test.

To investigate the systematic effect of the chords’ acoustical properties and the raters’ musical background upon the ratings, we entered all ratings of pleasantness and of roughness respectively as a dependent variable into two separate linear mixed-effects models (LMEs). Each LME had the participants’ *GMSI* scores and the stimuli’s *Parncutt* model predictions defined as fixed effects; it is the magnitude of these effects that we wished to quantify. On the other hand, *participant* and *stimulus* identity themselves were defined as random effects; thus, individual differences between participants, as well as between stimuli, are modelled (explained away) by assuming a different random intercept in the LME for each participant and for each stimulus. Interaction effects, as well as random slopes for the random effects, were not defined, in order to not have the model’s estimates penalised due to an increased number of factors, and as there was no particular hypothesis regarding how the effect of any one particular stimulus might be different for participants of different musical backgrounds.

## Results

### Rating distributions and averages

**Figure 1:**
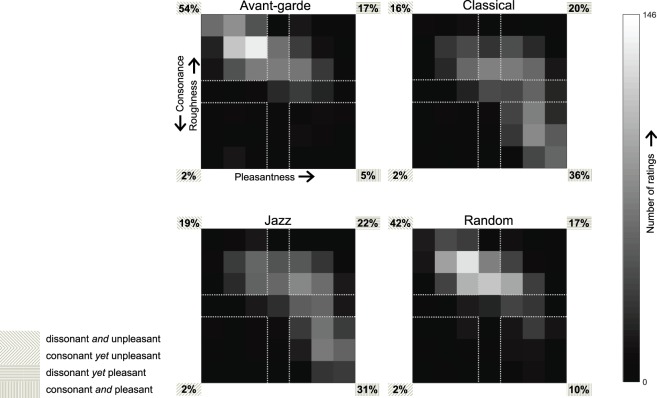
Histograms depicting the bivariate distribution of ratings across stimuli, separately for each style. The count of all rating pairs in each bin is indexed as per the greyscale bar; and clustered into the four corners of each rating space, where counts are expressed as a percentage of the total histogram mass. Italicised conjunctions (*and, yet*) in each cluster’s description reflect the working hypothesis that listeners generally ascribe a chord’s pleasantness in proportion to its degree of consonance.

To gain an overview of the distribution of – and relationship between – ratings across each style’s chords, the rating pairs corresponding to all exemplars in each style were represented in a two dimensional pleasantness-roughness rating space. This resulted in the 7 × 7 bivariate histograms plotted in Fig. 1, where the number of exemplars (from both repetitions and all participants) that received a certain rating pair is counted in each square (bin) of a histogram, as indexed in the greyscale bar. Rating spaces were then divided into the four clusters that result when binarising each dimension while excluding the median scores (middle rows & columns). For each style, each cluster’s cumulative count is then expressed as a proportion of the histogram’s total mass.

**Figure 2:**
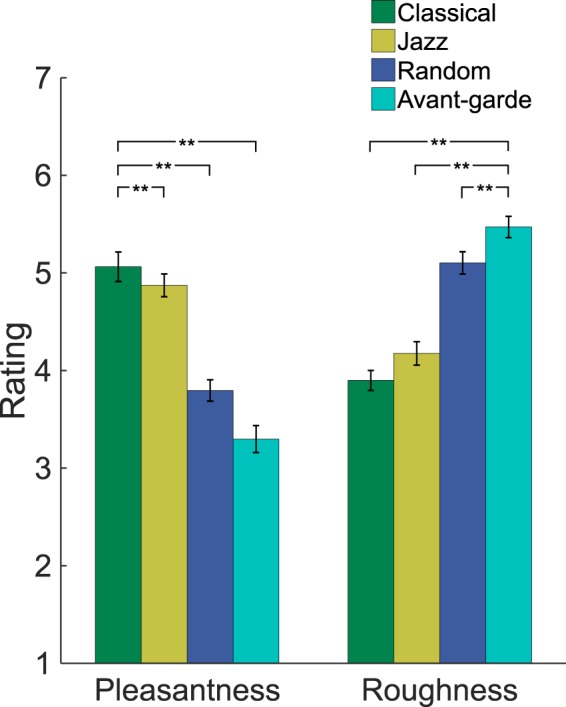
Mean pleasantness and roughness ratings for chords. Ratings are averaged across repetitions, exemplars and participants. Error bars indicate ±1SEM across participants. **p < 0.01.

Mean pleasantness and roughness ratings are shown in Fig. 2, broken down by style. All ratings were entered into a Rating Type (pleasantness/roughness) × Style (Classical/Jazz/Random/Avant-garde) repeated-measures ANOVA. The 2-way interaction was significant (F(3,87) = 203.42, *p* < 0.0001, η_p_^2^ = 0.82), as was the overall effect of Style for follow-up contrasts within each rating type (F(3,26) = 56.04, *p* < 0.0001, η_p_^2^ = 0.51; and F(3,26) = 87.57, *p* < 0.0001, η_p_^2^ = 0.69 respectively); this justified defining pair-wise contrasts which revealed significant differences between any two styles, for each rating type (all *p*s < 0.005). Thus, the ranking of the styles was Classical < Jazz < Random < Avant-garde for roughness, reversed for pleasantness. Although pleasantness ratings for Classical and Jazz stimuli correlated with GMSI score across participants (*r*(28) = 0.43, *p* < 0.05, Cohen’s *d* = 1.06; and *r*(28) = 0.51, *p* < 0.005, Cohen’s *d* = 1.38 respectively; uncorrected for multiple comparisons), all effects reported in the above ANOVA nonetheless remain significant even if GMSI is entered as a covariate to control for varying levels of musical sophistication across our sample, suggesting that this covariation was not single-handedly driving the effect.

### Sensory decoupling ability (SDA)

**Figure 3:**
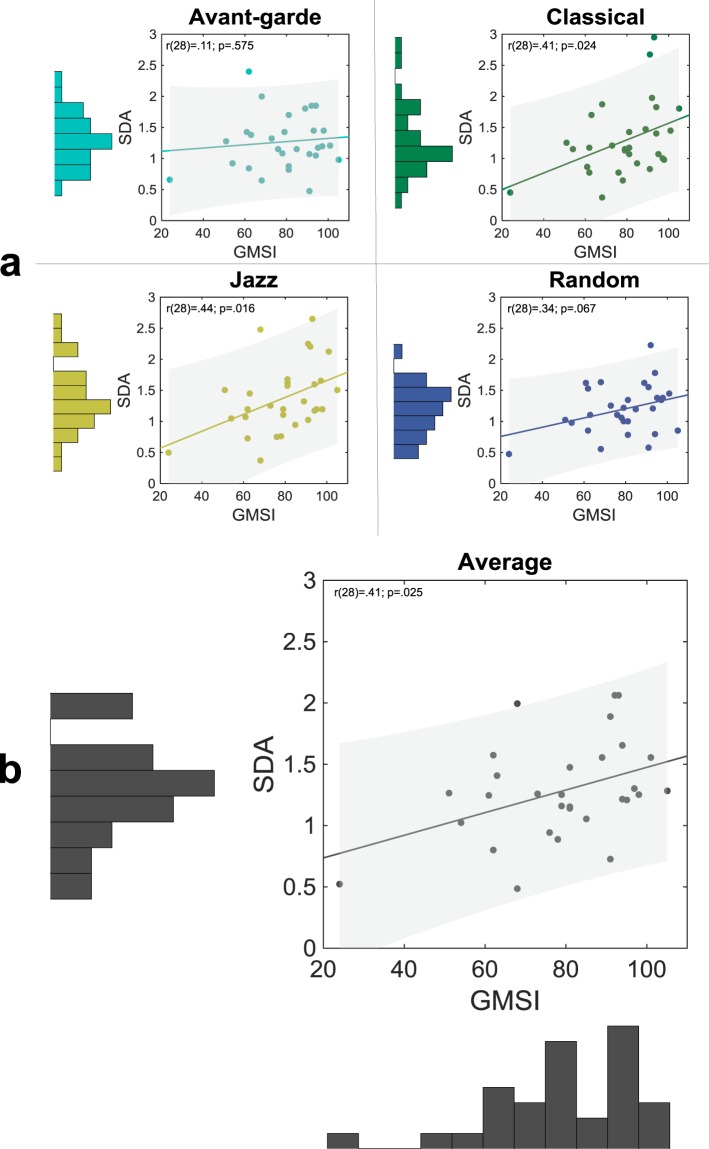
Cross-participant correlation between GMSI and sensory decoupling ability (SDA) scores, within styles (**a**) and collapsing across styles (**b**). Fit-line slopes are comparable across plots as measures of effect size, since axis ranges are matched. Each plot includes the least-squares regression line, with the 95% confidence band around it shaded.

Averaging across exemplars, repetitions and subjects, the average SDA ( ± σ) was 1.27 ± 0.43 for *Avant-garde*, *Classical* 1.29 ± 0.58, *Jazz* 1.37 ± 0.55, *Random* 1.20 ± 0.39. As depicted in Fig. 3, the correlation between the GMSI and SDA scores was significant for *Classical* and *Jazz* (*r*(28) = 0.41, 95% CI = [0.06, 0.67], *p* = 0.024; *r*(28) = 0.44, 95% CI = [0.09, 0.69], *p* = 0.016), as well as when collapsing SDA scores across styles (*r*(28) = 0.41, 95% CI = [0.06, 0.67], *p* = 0.025); it approached significance for *Random* (*r*(28) = 0.34, 95% CI = [−0.02, 0.62], *p* = 0.067); and it was non-significant for *Avant-garde* (*r*(28) = 0.11, 95% CI = [−0.26, 0.45], *p* = 0.58).

### Parncutt model fit

Correlations were significant, if weak, between ratings of roughness and corresponding values predicted by the Parncutt^18^ model, for all styles: *r*(598) = 0.21 for Avant-garde, 0.40 for Classical, 0.14 for Jazz, and 0.16 for Random; all *p*s < 0.001. Individual participants’ goodness-of-fit (within-subject *r* values) for the Parncutt model were not predicted by these participants’ GMSI scores, for any of the styles as well as when collapsing across styles (all *p*s > 0.2).

### Mixed-effects model

The LME model was used to investigate the systematic effect of both the chords’ acoustical properties and the raters’ musical background upon the ratings; the model estimation output is given in Table 1. The fixed effect of the Parncutt model score was significant for both ratings (all *p*s < 0.001). The trend was negative for pleasantness and positive for roughness, which confirms the significant correlations reported in section 4.3. The effect of GMSI was only significant for pleasantness, again confirming the same correlation reported earlier for classical and jazz stimuli. The random intercepts for both participant and stimulus were significant for both the pleasantness and the roughness LME (all *p*s < 0.05), confirming that there were individual differences – between participants and between stimuli – in these ratings.

**Table 1:** Mixed-effects modelling parameter estimates for pleasantness and of roughness ratings predicted via a participant’s GMSI score and a stimulus’ Parncutt model prediction.

		Pleasantness	Roughness
Model fit	AIC	7066.8	7049.8
		**Mean estimated coefficients**	
Fixed effects	(Intercept)	4.7822***	2.9961 ***
	Parncutt	−4.7474***	4.7557***
	GMSI	0.0119*	0.0025
		**SD (95% CI)**	
Random effects	(Participant intercept)	0.5650* (0.4340, 0.7357)	0.5230* (0.4011, 0.6822)
	(Stimulus intercept)	0.8688* (0.7388, 1.0217)	0.76767* (0.6515, 0.9045)
	Residual	0.9959* (0.9673, 1.0254)	0.99726* (0.9687, 1.0268)

AIC, Akaike information criterion; SD, standard deviation; CI, confidence interval; *p < 0.05, ***p < 0.001.

## Discussion

This study examined the relationship between ratings of sensory dissonance and pleasantness given to “real-world” chords characteristic of several musical styles. Further, we tested how well these ratings are predicted by existing models of sensory dissonance on the one hand, and raters’ musical sophistication on the other. We found the highest pleasantness ratings for the Classical style, followed by Jazz, Random and Avant-garde, with the mirror-opposite relationship for the average roughness of these styles. The chord distribution in these rating spaces was qualitatively different between styles, reflecting different pleasantness-dissonance profiles conferred by each style, although there were similarities among them (namely between Jazz and Classical on the one hand, and Random and Avant-garde on the other). Parncutt’s model of sensory dissonance performed poorly in predicting roughness ratings, and these predictions did not correlate with participants’ musical sophistication index. This index did, however, predict the degree to which the two rating dimensions dissociated at the intra-subject level, and this held both within and across styles.

### Impact of the manipulations examined

Several conclusions can be drawn from these findings. That (i) the rating differences between styles remained significant when controlling for musical sophistication, and (ii) both chord- and subject-specific predictors were significant in the LME model, suggests that both stimulus-side and listener-side factors are integral to explaining the rating data. In other words, ratings are driven, at least in part, by a combination of a listeners’ expertise and of the music’s harmonic characteristics. The bivariate rating distributions suggest that each style employs different mechanisms to trade-off consonance for pleasantness. Evidently, in Jazz it is possible to make use of dissonance in order to elicit pleasantness, while for Classical chords, the former occurs at the expense of the latter. This dissociation is reflected in the higher average SDA for Jazz in comparison to Classical chords.

It seems therefore that musical pleasure may well be derived from a certain degree of sensory dissonance, depending on the stylistic provenance of a given sonority. Indeed, our Jazz ratings contained the highest proportion of stimuli characterised as “dissonant *yet* pleasant”, while, conversely, Classical contained most “consonant *and* pleasant” stimuli (see Fig. 1). Surveying the distributions of all four styles reveals a striking asymmetry between dissonance and consonance: while perceived dissonance in a given sonority may or may not be pleasant, perceived consonance is almost never (but for 2% of the chords in each style) associated with unpleasantness.

Musical sophistication, as indexed by the GMSI, correlated positively with our SDA measure. With the latter we attempted to quantify a participant’s disinclination to conflate pleasantness and smoothness in music, or in other words the propensity to rate these two dimensions independently of one another. The correlation described was only significant for the Classical and Jazz styles. However, the fact that significance remained even when collapsing across all styles, and that there was also a trend for Random chords, suggests that this relationship is style-independent. Indeed, it is intuitively appealing that listeners with more experience in music will have learnt to derive pleasure from music not merely based on its surface (acoustical) properties, but from several other dimensions as well, such as structural cues and culturally- or autobiographically-relevant connotations; and that this ability extends across musical styles, familiar or not. This result also confirms earlier reports of pleasantness and smoothness ratings for musical intervals that were correlated only in non-musician listeners^25^. Furthermore, it is reasonable to assume that, had our listeners been told what style each rated stimulus belonged to, this would have added a further (top-down) component determining their rating strategy for both dimensions, perhaps more strongly for more musically-sophisticated listeners; and that this component might even have dominated the previously-discussed ones (stimulus acoustics and participant musicianship).

Surprisingly, in comparison to Random chords, our Avant-garde chords contained a lower proportion of chords rated “consonant *and* pleasant”, and a higher proportion of “dissonant *and* unpleasant”. That consonance and pleasantness ratings for Avant-garde were lower even than Random suggests that, in this particular sphere of music, not only is dissonance by itself part of the style’s aesthetics, but that this could potentially also extend to the pleasantness dimension. In other words, a high pleasantness rating would not come about neither via increased consonance (as in Classical), nor via increased dissonance (as in Jazz), but the aesthetic concept of pleasantness itself would simply require redefinition for this style of music and would, as such, not be suitably compared with the same dimension as appraised in a different style. This result might, therefore, best be understood by reference to the peculiarities of the aesthetic ideals of the Avant-garde style, such as its strict avoidance of tonal grammar and chords.

### Predictive power of the sensory dissonance model considered, and alternative explanations

While we acknowledge that, in addition to beating and roughness, sensory dissonance may also be underpinned at the acoustical level by the (partly co-occurring) phenomenon of fusion/harmonicity^10,23^, we nonetheless chose to consider a roughness-based model of sensory dissonance^18^. In our data, the values computed by the Parncutt model only managed to (reasonably well) predict participants’ roughness ratings for Classical chords; that they do not for the other styles supports our hypothesis that this model of sensory dissonance does not reflect the subjective experience of dissonance for musical styles that deviate from classical norms, such as Jazz. Furthermore, the model’s predictive power was uncorrelated with participants’ musical background (GMSI), suggesting that the model’s inadequacy is not compensated by high levels of rater musical sophistication, and that a new model would be needed to link computed roughness with rated roughness (ideally, at all levels of musical sophistication). An assessment of the Parncutt model would surely ultimate hinge upon the cognitive level at which one assumes dissonance ratings are made: if we consider surface/acoustical features to prevail (due to any of the mechanisms previously referenced^37–40,44^), then the present data can be taken to imply that the model in question does not sufficiently capture such features; if, on the other hand, we assume factors of expertise and culture to prevail in the ratings (as others probably would^6^), then the very attempt to predict these on the basis of the output of any (not just Parncutt’s) acoustic model might be ill-conceived.

The heterogeneous relationship between our two rating dimensions could, alternatively, also be explained in terms of the *source dilemma hypothesis* recently proposed by Bonin *et al*., according to which “uncertainty in the number, identity or location of sound objects elicits unpleasant emotions by presenting the auditory system with an incoherent percept”^57^. In that study, Bonin *et al*. obtained pleasantness ratings for short melodies and their pitch-, timbre- and spatial-deviants; and proposed the source dilemma hypothesis as a broad theoretical framework to explain the variance in the ratings. As this framework also posits sensory dissonance as a contributor to the overall source dilemma, it would seem plausible that the relative contribution of sensory dissonance is in turn determined by the style of music and a listener’s level of musical sophistication, as our results suggest. More speculatively, it could be that musical sophistication makes a listener more robust to the source dilemma, for instance by increasing the processing fluency proposed to mediate aesthetic pleasure^58,59^. Namely, it would do so by making uncertainty in the auditory scene (due to the sensory dissonance born out of a chord’s spectral make-up) less likely to affect pleasantness ratings, thus increasing the SDA.

### Comparisons with past studies

Comparison of the present results with those of the recent Lahdelma & Eerola^53^ study reveals a number of interesting points. Both studies obtained – from Western listeners – ratings of roughness (smoothness) and pleasantness (preference) for single isolated chords in various categories, originating from the literature of various musical styles (in our case) or from music theory (triads, tetra-, penta-, and hexachords) in the case of Lahdelma & Eerola^53^. The objective roughness of the employed chords was computed in each study using Parncutt’s model^18^ and the MIR Toolbox^60^, respectively. As for the findings, the relationship between smoothness and pleasantness was similar in the two studies, in that the ranked averages of the two dimensions tended to be coupled. However, in Lahdelma & Eerola’s study, for certain chord categories – the minor ninth, major ninth, and minor seventh – these dimensions dissociated: high pleasantness ratings were in these cases associated with moderately low smoothness. A further factor dissociating the two dimensions was chord inversion, with respect to which smoothness varied (maximum ratings for root position chords, followed by first and second inversions) whereas pleasantness did not. In our case, the deviations from the two dimensions’ coupling were reflected in a distribution of ratings that deviated from the straight line that would have indicated a straightforward trade-off between smoothness and pleasantness (see Fig. 1). This deviation existed in all styles used in our study but was particularly strong for Jazz and Classical chords. Lahdelma & Eerola’s finding of *increased* pleasantness for *dissonant* chords was assumed to be due to these chords’ high levels of aggregate dyadic consonance^61^ and was not correlated with listeners’ musical sophistication; by contrast, in our study, this latter factor predicted a listener’s tendency towards the smoothness-pleasantness decoupling (which we termed SDA). While both that study and ours concluded that (moderate levels of subjective or objective) roughness need not imply a decrease in a chord’s pleasantness, our emphasis was on how this observation holds across different musical styles, and how it is mediated by the listener’s musical background.

### Study limitations

A plausible criticism of our current method might refer to the non-exhaustive procedure used for stimulus selection, which relied on a relatively small number of carefully selected chords that we felt chiefly constituted *representative* stimuli in each style. We believe, however, that this criticism is to some degree mitigated by the fact that, in our linear mixed-effects modelling of the response data, which confirmed the effects reported earlier, both participants and stimuli were entered as random effects; in so doing, it is acknowledged that any one sample of participants and stimuli is inevitably arbitrary and thus that we do not expect by-participant and by-stimulus variability to systematically explain-away variance in the dependent variable.

As a second limitation we would note that, in order to even consider using Avant-garde music in this study, one is forced to neglect a large portion of music that has been composed over the past 50 years or so. In addition, much of the music written over that time is focused largely on timbre, “the aspect of sound quality not defined by pitch, duration or intensity”^62^. For pieces that hinge upon timbre as opposed to harmony, a study examining the relationship between consonance/dissonance and pleasantness needs to include timbre as an important component^63,64^. By contrast, if harmony appears at all in Avant-garde music, it tends to be valued for its perceived brightness or darkness, thus contributing to “colour” and overall timbral effect^65^. As opposed to the spectrum from consonance to dissonance in pitch, Avant-garde music is often constructed around spectra ranging from noise to purity in timbre^66,67^.

Finally, musical sophistication was the sole listener-side factor considered in this study. However, another such factor could conceivably – and very plausibly – be cultural background. Since our sample was homogeneously Western, future studies could explicitly manipulate cultural background and examine its role in dissonance perception.

### Conclusion and future research

To conclude, the findings reported here suggest that the place a sonority occupies on the consonance/dissonance spectrum is by no means the sole factor predicting the pleasure it will elicit; and that the “positive” end of this spectrum does not, by itself, automatically imply pleasantness in any given musical style. Furthermore, the relationship between these two dimensions not only depends upon characteristics of the perceived sonority and the aesthetic ideals idiosyncratic to its musical style of provenance, but also upon the perceiver, and in particular their degree of musical sophistication. While roughness models may describe well ratings of sonorities representing music-theoretically defined categories, chords sampled from the real-world musical literature add complexity that require additional explanatory factors. To account for this complexity, new models are needed that are capable of explaining the ecological experience of listening. This entails maintaining a context-sensitive stimulus selection (as performed in the present study), but also, in addition, possibly presenting chords within their original musical context rather than in isolation.

## Electronic supplementary material

SupplInfo

stimuli as MP3

## Data Availability

The complete data set for this study, including the audio files corresponding to the stimuli, can be accessed online at the Open Science Framework public repository (https://osf.io/dj8w9/), as well as on the journal’s webpage as Supplementary Information.
