# Author Correction: The pleasantness of sensory dissonance is mediated by musical style and expertise

**DOI:** 10.1038/s41598-019-44427-5

**Published:** 2019-06-18

**Authors:** Tudor Popescu, Monja P. Neuser, Markus Neuwirth, Fernando Bravo, Wolfgang Mende, Oren Boneh, Fabian C. Moss, Martin Rohrmeier

**Affiliations:** 1 Institute for Art History and Musicology, TU Dresden, Dresden, Germany; 2 Department of Psychiatry and Psychotherapy, Eberhard Karls University Tübingen, Tübingen, Germany; 3 Digital Humanities Institute, École Polytechnique Fédérale de Lausanne, Lausanne, Switzerland; 4 Department of Music, University of California, Berkeley, USA; 5 Department of Neurology, Medical University of Vienna, Vienna, Austria

Correction to: *Scientific Reports* 10.1038/s41598-018-35873-8, published online 31 January 2019

The original version of this Article contained errors.

Affiliation 1 was incorrectly given as “Institute for Art History and Musicology, TU Dresden, Germany”. The correct affiliation is listed below:

Institute for Art History and Musicology, TU Dresden, Dresden, Germany

Furthermore, an affiliation for Tudor Popescu was omitted. The correct affiliations for Tudor Popescu are listed below:

Institute for Art History and Musicology, TU Dresden, Dresden, Germany

Department of Neurology, Medical University of Vienna, Vienna, Austria

Finally, the original version of the Acknowledgements was incomplete.

“We thank Daniel Harasim, Jonathan Walther and Devin B. Terhune for help with programming, stimulus creation and improvement of figures respectively. We also gratefully thank the Editorial Board of Scientific Reports and in particular the two anonymous reviewers, who provided very insightful commentary on a previous version of this manuscript. This research was carried out with funding from the Zukunftskonzept at TU Dresden (Grant No. ZUK 64), a part of the Exzellenzinitiative of the Deutsche Forschungsgemeinschaft (DFG).”

now reads:

“We thank Daniel Harasim, Jonathan Walther and Devin B. Terhune for help with programming, stimulus creation and improvement of figures respectively. We also gratefully thank the Editorial Board of Scientific Reports and in particular the two anonymous reviewers, who provided very insightful commentary on a previous version of this manuscript. This research was carried out with funding from the Zukunftskonzept at TU Dresden (Grant No. ZUK 64), a part of the Exzellenzinitiative of the Deutsche Forschungsgemeinschaft (DFG). Tudor Popescu, previously at TU Dresden and presently at the Medical University of Vienna, kindly acknowledges the support of Professor Roland Beisteiner and of the ‘Clinical fMRI’ research group.”

These errors have now been corrected in the HTML and PDF versions of the Article.

