# Supplementary material for: The pleasantness of sensory dissonance is mediated by musical style and expertise
Source: Sci Rep. 2019 Jan 31;9:1070. doi: 10.1038/s41598-018-35873-8 (PMC6355932; doi:10.1038/s41598-018-35873-8)
Supplement: Supplementary file 1 — SupplInfo [file 41598_2018_35873_MOESM1_ESM.pdf]

The pleasantness of sensory dissonance is mediated by musical style and expertise

Tudor Popescu, Monja P. Neuser, Markus Neuwirth, Fernando Bravo,
Wolfgang Mende, Oren Boneh, Fabian C. Moss, Martin Rohrmeier

Supplementary Information

Stimuli used (4 musical styles × 20 chords per style)

Classical

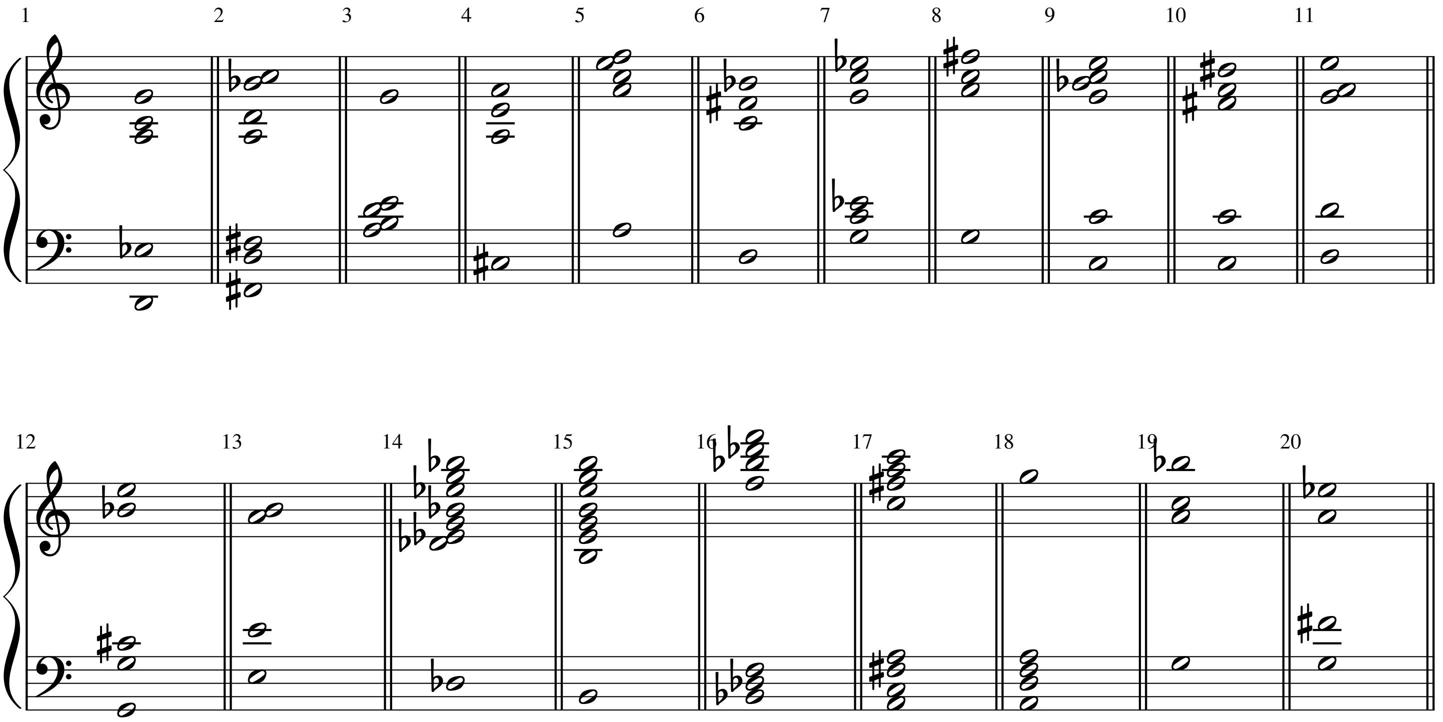

Avant-garde

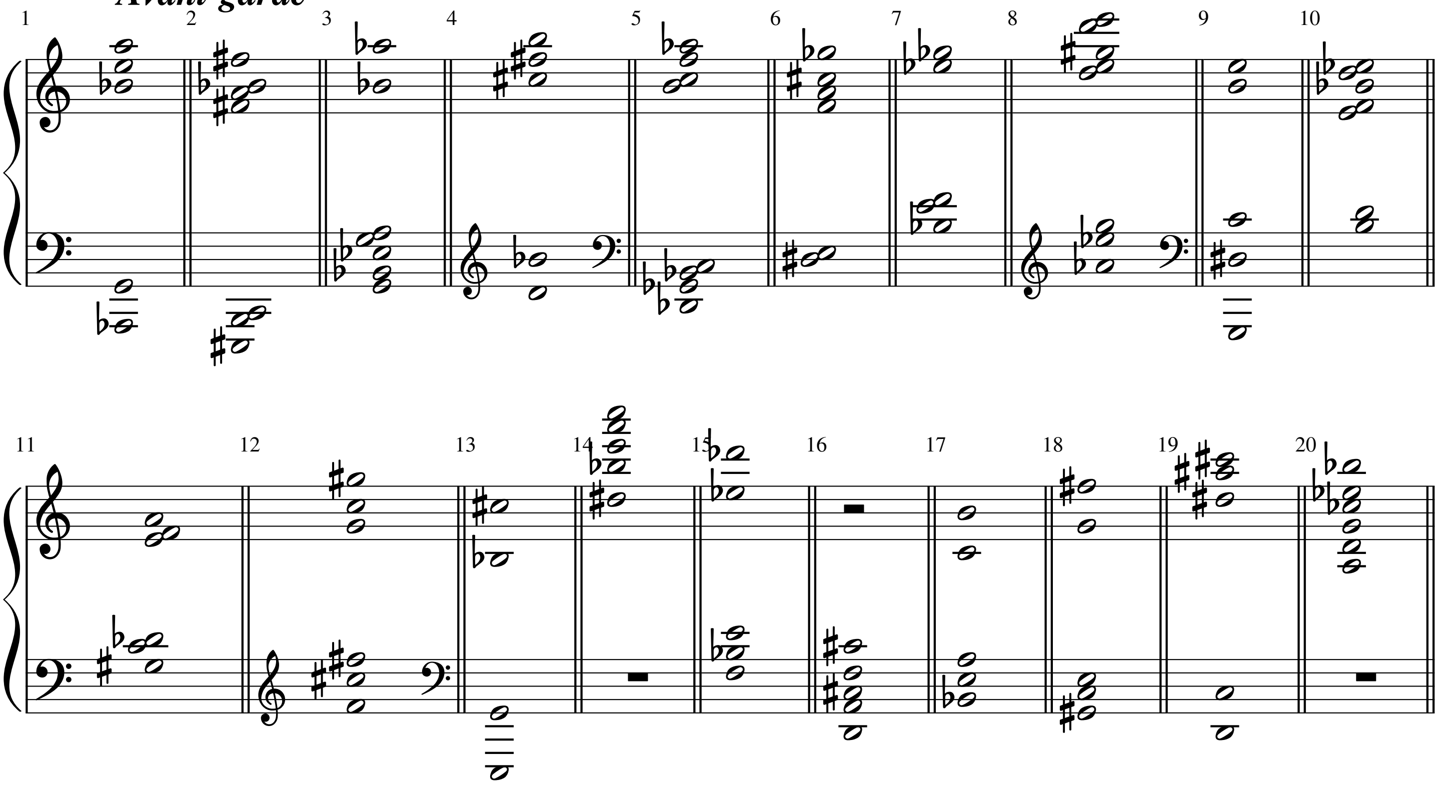

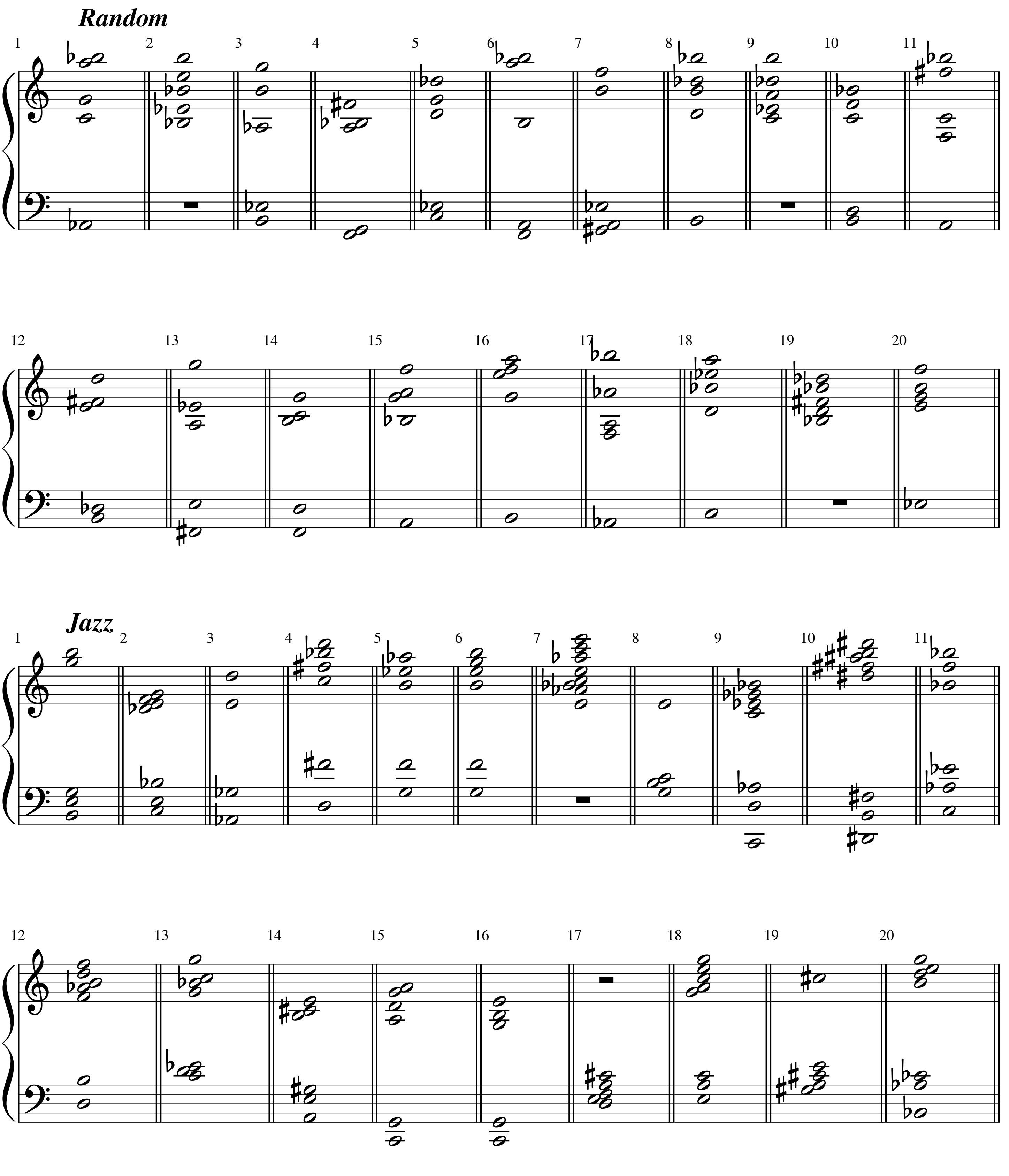
Random
Jazz

Chords' provenance

Classical

Stim.#	Composer	Work Title (mvt.=movement)	Measure	Beat
1	Beethoven	Piano sonata Op. 81a, 2nd mvt.	11	1
2	Beethoven	Piano sonata Op. 81a, 2nd mvt.	12	2
3	Schumann	Liederkreis, Op. 39, #4 "Die Stille"	7	4
4	Brahms	Piano Concerto No. 1, 1st mvt.	12	1
5	Beethoven	Symphony Op. 55, 1st mvt.	276	1
6	Mendelssohn	Songs without Words, Op. 53 No. 3	102	4
7	Mozart	Piano sonata K. 283, 3rd mvt.	163	1
8	Mozart	Piano sonata K. 283, 2nd mvt.	4	3
9	Beethoven	Symphony No. 1, 1st mvt.	1	1
10	Beethoven	String Quartet Op. 18 No. 1, 2nd mvt.	52	2
11	Mozart	Requiem, Dies Irae	2	1
12	Mozart	Requiem, Dies Irae	7	3
13	Mozart	Requiem, Dies Irae	18	3
14	Haydn	Keyboard Trio Hob. XV:26, 1st mvt.	47	2.5
15	Haydn	Keyboard Trio Hob. XV:26, 1st mvt.	49	2.5
16	Schubert	Wandererfantasie, D. 760, 1st mvt.	134	1
17	Clementi	Piano sonata, Op. 40 No. 1, 1st mvt.	5	4
18	Clementi	Piano sonata Op. 40 No. 1, 1st mvt.	28	1
19	Clementi	Piano sonata Op.50 No. 3, 3rd mvt.	1	1
20	Clementi	Piano sonata Op.50, No. 3, 3rd mvt.	3	1

Avant-garde

Stim.#	Composer	Work Title (mvt.=movement)	Page	Line	Edition
1	Scelsi	Piano Sonata No. 3	10	penultimate	Paris: Editions Salabert (1987)
2	Scelsi	Piano Sonata No. 4	6	penultimate	
3	Scelsi	Piano Sonata No. 3	12	1	
4	Berio	6 encores	3	3	Wien: Universal Edition (1990)
5	Berio	6 encores	17	3	
6	Feldman	Piano Piece to Philip Guston	1	1	New York: C.F. Peters Corporation (1963)
7	Feldman	Piano Piece to Philip Guston	1	2	
8	Sciarrino	De la nuit	8	1	Milano: G. Ricordi (1971)
9	Boulez	Sonata for piano No. 2	10	2	Paris : Heugel (1950)
10	Xenakis	A.R. (Hommage à Ravel)	1	3	Paris : Editions Salabert (1989)
11	Xenakis	A.R. (Hommage à Ravel)	1	bottom	
12	Boulez	Sonata for piano No. 2	3	3	Paris : Heugel (1950)
13	Boulez	Sonata for piano No. 2	21	last	
14	Schoenberg	Five Pieces, Op. 23, No. 1	3	33	Kopenhagen: Wilhelm Hansen (1923)
15	Schoenberg	Five Pieces, Op. 23, No. 3	3	4	
16	Webern	Five Songs, Op. 3, No. 1 "Dies ist ein Lied für dich allein"	1	4	Wien: Universal Edition (1921)
17	Webern	Five Songs, Op. 3, No. 1 "Dies ist ein Lied für dich allein"	1	2	
18	Xenakis	Herma	7	penultimate	Boosey and Hawkes Music Publishers (1967)
19	Xenakis	Herma	10	3	
20	Ustvolskaya	Sonata No. 4, mvt. 2	4	3	Hamburg: H. Sikorski (1996)

Jazz

Stim.#	Page	Line	Measure	Edition
1	5	2	3	Jazz. Milwaukee, WI: Hal Leonard. ISBN 978-0-634-05410-5
2	10	1	1	
3	11	3	2	
4	20	4	2a	
5	20	4	2b	
6	23	5	2	
7	24	5	3	
8	26	2	1	
9	26	4	2	
10	31	4	2	
11	55	1	3	
12	55	3	4	
13	56	2	4	
14	56	4	3	
15	105	1	3	
16	106	1	2	
17	107	3	1	
18	113	3	3	
19	147	4	2	
20	157	2	2	
